# Predictive value of the time interval between embryo loading and transfer for IVF/ICSI success: a prospective cohort study

**DOI:** 10.1186/s12958-015-0048-6

**Published:** 2015-05-29

**Authors:** Kazem Nouri, Clemens B. Tempfer, Katharina Walch, Regina Promberger, Sema Dag, Johannes Ott

**Affiliations:** Department of Obstetrics and Gynecology, Clinical Department of Gynecologic Endocrinology and Reproductive Medicine, Medical University of Vienna, Waehringer Guertel 18-20, 1090 Vienna, Austria; Department of Surgery, Medical University of Vienna, Waehringer Guertel 18-20, 1090 Vienna, Austria; Department of Obstetrics and Gynecology, Ruhr University Bochum, Bochum, Germany; Marienhospital Herne, Hoelkeskampring 40, 44625 Herne, Germany

**Keywords:** Embryo transfer, Catheter, Embryo loading time, Pregnancy rate, Live birth rate

## Abstract

**Background:**

The influence of embryo loading time (ELT) and the time interval between embryo loading and embryo transfer (TIEL-ET) on the success of IVF/ICSI is unknown.

**Methods:**

In a prospective cohort study, we aimed to ascertain the influence of ELT and TIEL-ET on ongoing pregnancy rate (OPR) and life birth rate (LBR). Data from 603 consecutive embryo transfers between January 2008 and December 2013 were collected. A complete data set including the outcomes of interest OPR and LBR was available for 410 women. The primary outcome was IVF/ICSI success, defined as OPR and LBR.

**Results:**

We used univariate and multivariate logistic regression for analysis. In a multivariate analysis, age (odds ratio [OR] 0.94; 95 % confidence interval [CI] 0.89–0.99), catheter type (OR 0.45; 95 % CI 0.24–0.84), and uterine length (OR 1.03; 95 % CI 1.01–1.06), but not ELT and TIELT-ET were independently associated with OPR. Regarding LBR, age (OR 0.93; 95 % CI 0.88–0.98), catheter type (OR 0.41; 95 % CI 0.22–0.79), and uterine length (OR 1.03; 95 % CI 1.01–1.06), but not ELT and TIELT-ET were independent predictors.

**Conclusion:**

We conclude that speed of embryo transfer is not critical for the success of IVF/ICSI. However, care should be taken to choose catheter types proven to be associated with a high success rate.

## Background

The embryo transfer (ET) is an integral part of IVF. Technically, ET is an easy procedure, but it requires a well-standardized sterile setting, a well-trained staff, and careful handling of the instruments during embryo aspiration and catheter insertion into the uterus [1]. Several factors before, during and after ET have been examined regarding their influence on the ongoing pregnancy rate (OPR) and the life birth rate (LBR). For example, in a systematic review and meta-analysis of ten randomized trials in 4141 women, Abou-Setta et al. demonstrated that using soft catheters for ET resulted in a significantly higher pregnancy rate compared to firm catheters [2]. In addition, experience of the physician, use of ultrasound guidance, subjective ease of the procedure, and absence of blood on the catheter were demonstrated by others to increase the subsequent pregnancy rates [3–6]. Regarding the site of embryo deposition, a meta-analysis of six trials showed a positive trend towards a higher OPR and LBR when ET was performed into the lower half of the uterine cavity [7]. Other factors do not seem to have an impact on the success of ET. For example, in a randomized trial of 102 women undergoing IVF-ET, loading of air into the catheter to bracket the embryo-containing medium did not alter implantation rates or OPR [8]. In contrast to the above mentioned factors, less attention has been paid to the prognostic impact of the procedural speed used to perform ET. The only study looking at this aspect of ET was performed by Matorras et al. and measured the time interval between embryo catheter loading and discharging in 450 consecutive fresh cycles [9]. The authors found that the longer the interval between loading and discharging the embryos, the lower the pregnancy and implantation rates. Specifically, OPR broken down by the duration of the time interval between catheter loading and discharging were as follows: 39 % for a time interval <30 s, 33 % for a time interval between 31 and 60 s, 32 % for a time interval between 61 and 120 s, and 19 % for a time interval >120 s. These data indicate that there is only a gradual decrease in the OPR until a time interval of 120 s has been reached, but a sharp decrease of OPR when >120 s are needed for the procedure. The time interval assessed in this study has essentially two parts, namely the embryo loading time (ELT) and the time interval between embryo loading and the end of the embryo transfer (TIEL-ET). Since ELT and TIEL-ET are two different procedures, mostly performed by different people, i.e., a technician and a physician, it is conceivable that they might have an independent effect. The specific influence of ELT and TIEL-ET on the success of IVF/ICSI has not been prospectively investigated. Therefore, we performed a prospective cohort study with predefined starting and stopping points investigating the separate effects of ELT and TIEL-ET on OPR and LBR in first-time, fresh ETs.

## Methods

This study was conducted at the Division of Gynecologic Endocrinology and Reproductive Medicine, Department of Obstetrics and Gynecology, Medical University of Vienna, Austria, between January 2008 and December 2013. All consecutive women who underwent their first IVF/ICSI cycle were eligible for the study and their data were prospectively collected. The study was approved by the ethics committee of the Medical University of Vienna (project number 1046/2014). Written informed consent was obtained from all study participants. Inclusion criteria were the use of a standard antagonist or agonist protocol, age <42 years, regular menstrual cycles every 25–35 days, basal FSH levels <12 mIU/mL at day 3 of the menstrual cycle, and a normal uterine cavity on transvaginal ultrasound on day 2 of the menstrual cycle. Moreover, only women with transfer of two fresh embryos from a fresh cycle were included. Women with transfer of cryoconserved embryos were excluded.

### IVF treatment

All women were treated using an antagonist or agonist protocol. Ovarian stimulation was started with 150–200 IU of recombinant follicle-stimulating hormone (FSH) (Puregon®, MSD Pharma, Austria or Gonal F®, Serono, Austria). Antagonists (Orgalutran®, MSD Pharma, Austria or Cetrotide®, Serono, Austria) were given when the leading follicle reached a diameter of 12 mm. Women treated with an agonist protocol underwent standard down-regulation with a GnRH agonist (Decapeptyl®, Ferring, Austria) on day 21 of the preceding cycle. After down regulation, defined as E2 less than 50 pg/ml, stimulation was started in analogy to the antagonist protocol. Follicle monitoring was performed by transvaginal sonography. When necessary, the FSH dosage was adjusted according to the follicle numbers and diameters. When adequate stimulation was achieved (≥3 follicles of at least 18 mm in diameter), 10,000 IU of human chorionic gonadotropin (hCG) (Pregnyl®, AESCA Pharma, Austria) were administered. Oocyte retrieval was performed 35 h after hCG injection. Conventional IVF following standard techniques was used for fertilization. A maximum of two embryos were transferred between days 3 and 5 after oocyte retrieval. Almost all women received luteal support with vaginal progesterone (Utrogestan®, Meda Pharma GmbH, Austria) 600 mg per day with a few exceptions of women who preferred oral dydrogesterone (Duphaston®, Abbott GmbH, Austria) 20 mg per day.

### Embryo transfer technique

ET was performed on day 3 or day 5 after oocyte retrieval. The decision was made according to the quantity and quality of the embryos. A maximum of two embryos were inserted. Before ET, patients underwent transvaginal sonography. All patients were in a lithotomy position and were requested to keep their bladder full. Patients received magnesium carbonate (Magnosolv Granulat®, Meda Pharma, Austria) orally within 1 h before ET. Transabdominal sonography was performed prior to and during the procedure to monitor the catheter placement in the uterine cavity. A sterile speculum (bivalve speculum) was inserted gently into the vagina and was opened to expose the exocervix, which was cleaned with a sterile cotton swab soaked in Ham’s F-10 medium by the physician. The uterus and the uterine cavity were visualized on transabdominal sonography. Embryos were loaded by a biomedical assistant starting with flushing of the transfer catheter with embryo culture medium followed by suctioning of 20 μl of culture medium into the catheter, then 20 μl of culture medium containing the embryos and lastly, and 5 μl of air on the tip of the catheter to bracket the embryo-containing medium. ELT was defined as the time interval between the start of flushing the transfer catheter with embryo culture medium and the end of the aspiration of the embryo into the catheter. ELT was measured by an independent observer using a stop watch. TIEL-ET was defined as the time interval between the end of ELT and the retraction of the catheter from the uterus and was measured by the treating physician using a stop watch. Specifically, the stop watch was started when the loaded catheter was passed by the technician to the physician. The catheter was inserted into the lower uterine segment until the outer sheath of the catheter was at the lowest point of the endometrial cavity. At this point, no further movement of the outer catheter was carried out. The inner catheter was slowly advanced with the aim of placing the embryos in the middle of the uterine cavity, followed by a 45° rotation and removal of the catheter. This was defined as the end of TIEL-ET. Finally, the physician handed the catheter to the technician in order to check if any embryos were retained. Two types of catheters were used at the discretion of the physician: the Wallace embryo transfer catheter (Wallace Ltd, Colchester, UK), a soft catheter, and the Labotect embryo transfer catheter (Hytek Scientifics Inc., Goettingen, Germany), a rigid catheter.

### Parameters analyzed

The main outcome parameter of this study was success of IVF/ICSI, defined as ongoing pregnancy rate (OPR) and life birth rate (LBR). OPR was defined as positive heartbeat of an embryo in the uterine cavity in the fifth week after ET. LBR was defined as delivery of a living fetus ≥500 g. The position of the uterus (anteverted/midposed/retroverted), the uterocervical angle (normal: 120–180°; pointed angle: 90–120°; very pointed angle: <90°), and the length and width of the uterus were measured by transvaginal sonography according to Sallam et al. [10]. The catheter type was recorded.

### Statistical analysis

Nominal variables are reported as numbers and frequencies, continuous variables as means and standard deviation of the mean. A multivariable logistic regression model was used with OPR and LBR as the dependent variables and ELT, TIEL-ET, age, body mass index (BMI), catheter (soft [Wallace] vs. rigid [Labotect]), substance used for ovulation induction, uterine position, uterine angle, uterine length, endometrial thickness, and endometrial length as the dependent variables. Odds ratios (OR) and 95 % confidence interval (CI) were used to describe the results of logistic regression. *P*-values <0.05 were considered statistically significant. Statistical analyses were performed with the SPSS software package, version 19 (SPSS, Chicago).

## Results

Six hundred three consecutive women undergoing their first IVF/ICSI cycle were recruited between January 2008 and December 2013. One hundred ninety three women were excluded because information about IVF/ICSI success was not available (*n* = 45) or controlled ovarian stimulation and oocyte retrieval were not followed by ET in the same treatment cycle (*n* = 148). Therefore, a complete data set including the recorded times of ELT and TIEL-ET and the outcomes of interest OPR and LBR was available for 410 women and was used for analysis. Patient characteristics of these 410 patients are given in Table 1. In the complete study cohort, OPR and LBR were 27.3 and 23.9 %, respectively. The soft catheter was used in 74/410 (18.0 %) patients and the rigid catheter in 336/410 (82.0 %) patients. ET was achieved at first attempt in 405/410 (98.8 %) patients. Both catheter types were equally successful (72/74 [97 %] vs 333/336 [99 %]; *p* = n.s.). In 5/410 (1.2 %) patients, the first attempt failed (soft catheter: *n* = 2; rigid catheter: *n* = 3). In all five cases, a rigid catheter was used for the second attempt and was successful in all cases.

**Table 1:** Patient characteristics

	All patients	Patients with rigid catheter	Patients with soft catheter	*p*
Number of patients	410	336	74	-
Age (years)^a^	31.7 ± 5.3	31.7 ± 5.2	31.7 ± 5.5	0.9
Body mass index (kg/m^2^)^a^	24.6 ± 5.5	24.6 ± 5.6	24.0 ± 5.5	0.4
Tubal factor^b^	75 (18.3)	60 (17.9)	15 (20.3)	0.6
Male factor^b^	285 (69.5)	229 (68.2)	56 (75.7)	0.2
Endometriosis^b^	69 (16.8)	58 (17.3)	11 (14.9)	0.6
Polycystic ovary syndrome^b^	44 (10.7)	34 (10.1)	10 (13.5)	0.4
Idiopathic infertility^b^	9 (2.2)	5 (1.5)	4 (5.4)	0.06
Number of retrieved oocytes^a^	4.8 ± 3.3	4.5 ± 3.1	5.0 ± 3.5	0.6
Length of stimulation (days)^a^	11 (10–12)	11 (10–12)	11 (10–12)	0.9
ICSI^b^	145 (35.4)	112 (33.3)	30 (40.5)	0.2
Ongoing pregnancy rate^b^	112 (27.3)	84 (25.0)	28 (37.8)	0.02
Live birth rate^b^	98 (23.9)	64 (19.0)	24 (32.4)	0.01

Data are provided as ^a^means ± standard deviation and ^b^ *n* (%)

Table 2 shows the results of a univariate and multivariate analysis of ELT, TIEL-ET, age, BMI, catheter type, substance used for ovulation induction, uterine position, uterine angle, uterine length, endometrial thickness, and endometrial length as predictors of OPR. In this analysis, only age (OR 0.94; 95 % CI 0.89–0.99), catheter type (OR 0.45; 95 % CI 0.24–0.84), and uterine length (OR 1.03; 95 % CI 1.01–1.06) were independent predictors of OPR. Of note, ELT and TIELT–ET were both not associated with OPR in the univariate and multivariate analysis. Patient characteristics such as age, BMI, number of retrieved oocytes, length of stimulation days, and the percentage of women with tubal factor, male factor, endometriosis, polycystic ovary syndrome, idiopathic infertility, and ICSI were not significantly different between women in whom soft and rigid catheters were used (Table 1). In addition, we compared ELT and TIEL-ET in women with day 3 transfer and day 5 transfer. No statistically significant differences were ascertained (40.6 ± 19.1 vs. 44.1 ± 22.6, *p* = 0.07 and 57.3 ± 19.6 vs. 60.0 ± 25.5, *p* = 0.6, respectively).

**Table 2:** Multivariate logistic regression model of factors associated with ET for the prediction of ongoing pregnancy

		Pregnancy	No pregnancy	Univariate analysis			Multivariate analysis		
		(*n* = 112)	(*n* = 298)	OR	95 % CI	LR test	OR	95 % CI	LR test
Age (years)^a^		30.4 ± 5.0	32.2 ± 5.3	0.94	(0.90;0.98)	0.008	0.94	(0.89;0.99)	0.013
BMI (mg/m^2^)^a^		24.1 ± 4.8	24.7 ± 5.8	0.98	(0.94;1.02)	0.347	-	-	-
Catheter type^b^		84 (75.0)	254 (85.2)	0.52	(0.30;0.90)	0.02	0.45	(0.24;0.84)	0.013
Time interval between oocyte retrieval and ET (days)^a^		3.5 ± 1.2	3.1 ± 1.3	1.23	(1.03;1.47)	0.02	1.16	(0.94;1.43)	0.169
Substance used for ovulation induction	hCG^b^	89 (79.5)	237 (79.5)	Reference	Reference	0.558	-	-	-
	Synthetic GnRH^b^	3 (2.7)	13 (4.4)	0.45	(0.10;2.05)		-	-	
	Choriogonadotropin alpha^b^	20 (17.6)	48 (16.1)	2.12	(1.16;3.86)		-	-	
ELT (seconds)^a^		42.5 ± 19.8	40.1 ± 18.5	1.01	(0.99;1.02)	0.314	-	-	-
TIEL-ET (seconds)^a^		56.5 ± 21.9	60.0 ± 25.4	1.00	(0.99; 1.00)	0.496	-	-	-
Uterine position	Anteverted^b^	94 (83.9)	261 (87.6)	Reference	Reference	0.144	-	-	-
	Midposed^b^	14 (12.5)	26 (8.7)	1.85	(0.88;3.89)		-	-	
	Retroverted^b^	2 (1.8)	11 (3.7)	0.33	(0.04;2.67)		-	-	
Uterine angle (Corpus–Cervix)	Normal^b^	77 (68.8)	178 (59.7)	Reference	Reference	0.108	-	-	-
	Pointed^b^	27 (24.1)	54 (18.1)	1.22	(0.67;2.22)		-	-	
	Very pointed^b^	8 (7.1)	66 (22.1)	0.42	(0.17;1.05)		-	-	
Uterine length (mm)^a^		85.2 ± 12.4	80.7 ± 40.9	1.03	(1.01;1.05)	0.015	1.03	(1.01;1.06)	0.006
Endometrial thickness (mm)^a^		12.1 ± 3.5	11.4 ± 2.7	1.05	(0.99;1.12)	0.125	-	-	-
Endometrial length (mm)^a^		29.1 ± 8.1	28.6 ± 7.4	1.01	(0.97;1.04)	0.640	-	-	-

*ET* embryo transfer, *OR* odds ratio, *CI* confidence interval, *LR* test likelihood ratio test, *hCG* human chorion gonadotropin, *GnRH* gonadotropin releasing hormone, *ELT* embryo loading time, *TIEL-ET* time interval between embryo loading and ET

Data are provided as ^a^mean ± standard deviation or ^b^ *n* (%)

Table 3 shows the results of a univariate and multivariate analysis of ELT, TIEL-ET, age, BMI, catheter type, substance used for ovulation induction, uterine position, uterine angle, uterine length, endometrial thickness, and endometrial length as predictors of LBR. In accordance with the results for OPR, only age (OR 0.93; 95 % CI 0.88–0.98), catheter type (OR 0.41; 95 % CI 0.22–0.79), and uterine length (OR 1.03; 95 % CI 1.01–1.06), but not ELT and TIELT-ET were independent predictors of LBR. Using Spearman’s rank correlation, we found that ELT and TIELT-ET were significantly correlated (*r* = 0.557; *p* < 0.001). Thus, a speedy ELT was usually followed by a speedy TIEL-ET, whereas a slow ELT was usually followed by a slow TIEL-ET. Because catheter type was independently associated with OPR and LBR, we further assessed this variable. Using a soft catheter resulted in an OPR of 38.9 % (28/72) whereas using a rigid catheter resulted in a lower OPR (24.8 % [84/338]). This difference was statistically significant (*p* = 0.02). In accordance with this finding, LBR was also significantly higher when a soft catheter was used (34.7 % [25/72] vs 21.6 % [73/338]; *p* = 0.02). Of note, ELT was significantly longer when the soft catheter was used (50.0 ± 19.8 for the soft catheter vs. 39.4 ± 18.5 s for the rigid catheter; *p* = 0.001). In contrast, there was no difference in TIEL-ET between the soft and rigid catheter (63.4 ± 41.3 s vs 58.3 ± 48.1 s, respectively; *p* = 0.3).

**Table 3:** Multivariate logistic regression model of factors associated with ET for the prediction of life birth

		Life birth	No life birth	Univariate analysis			Multivariate analysis		
		(*n* = 98)	(*n* = 312)	OR	95 % CI	LR test	OR	95 % CI	LR test
Age (years)^a^		30.2 ± 5.1	32.2 ± 5.2	0.93	(0.89;0.97)	0.002	0.93	(0.88;0.98)	0.011
BMI (mg/m^2^)^a^		24.2 ± 5.0	24.7 ± 5.7	0.99	(0.94;1.03)	0.561	-	-	-
Catheter type^b^		64 (65.3)	274 (87.8)	0.48	(0.27;0.85)	0.011	0.41	(0.22;0.79)	0.008
Time interval between oocyte retrieval and ET (days)^a^		3.5 ± 1.1	3.1 ± 1.3	1.28	(1.10;1.54)	0.009	1.17	(0.94;1.47)	0.126
Substance used for ovulation induction	hCG^b^	74 (75.5)	252 (80.8)	Reference	Reference	0.424	-	-	-
	Synthetic GnRH^b^	3 (2.7)	13 (4.4)	0.58	(0.13;2.64)		-	-	
	Choriogonadotropin alpha^b^	21 (21.4)	47 (15.1)	1.38	(0.75;2.54)		-	-	
ELT (seconds)^a^		42.6 ± 20.6	40.2 ± 18.3	1.01	(0.99;1.02)	0.343	-	-	-
TIEL-ET (seconds)^a^		54.2 ± 23.0	57.8 ± 24.3	1.00	(0.99; 1.00)	0.506	-	-	-
Uterine position	Anteverted^b^	82 (83.7)	273 (87.5)	Reference	Reference	0.391	-	-	-
	Midposed^b^	13 (13.3)	27 (8.7)	1.52	(0.69;3.34)		-	-	
	Retroverted^b^	1 (1.0)	12 (3.8)	0.40	(0.053.26)		-	-	
Uterine angle (Corpus–Cervix)	Normal^b^	68 (69.4)	187 (59.9)	Reference	Reference	0.259	-	-	-
	Pointed^b^	23 (23.5)	58 (18.6)	1.28	(0.68;2.41)		-	-	
	Very pointed^b^	7 (7.1)	67 (21.5)	0.55	(0.22;1.37)		-	-	
Uterine length (mm)^a^		85.3 ± 12.6	80.8 ± 40.7	1.02	(1.01;1.05)	0.020	1.03	(1.01;1.06)	0.007
Endometrial thickness (mm)^a^		12.2 ± 3.6	11.7 ± 2.7	1.01	(0.97;1.04)	0.602	-	-	-
Endometrial length (mm)^a^		29.2 ± 7.7	28.6 ± 7.6	1.06	(0.99;1.13)	0.640	-	-	-

*ET* embryo transfer, *OR* odds ratio, *CI* confidence interval, *LR* test likelihood ratio test, *hCG* human chorion gonadotropin, *GnRH* gonadotropin releasing hormone, *ELT* embryo loading time, *TIEL-ET* time interval between embryo loading and ET

Data are provided as ^a^mean ± standard deviation or ^b^ *n* (%)

## Discussion

In a prospective cohort study of 410 consecutive women undergoing their first IVF/ICSI, we found no association between ELT and TIEL-ET and OPR and LBR. In our study cohort, OPR and LBR were 27.3 and 23.9 %, respectively and they did not vary significantly by the duration of ELT and TIEL-ET. This is the main finding of our study demonstrating that the procedural speed of ELT as well as the procedural speed of TIEL-ET do not affect the success of IVF/ICSI. Fast as well as slow ELT and TIEL-ET are both efficient and there is no need to implement specific time benchmarks for these procedures.

In a multivariate logistic regression model, patient age, uterine length, and catheter choice significantly affected both OPR and LBR. Specifically, a longer uterus increased the chance of an ongoing pregnancy with an OR of 1.03, whereas the use of a rigid catheter compared with a soft catheter reduced the chance of an ongoing pregnancy with an OR of 0.45. These findings are in accordance with previously published data in the literature. For example, Egbase et al. prospectively assessed uterine length in 807 ETs and found that the highest implantation and clinical pregnancy rates were seen in women with a cavity length of 7–9 cm, whereas longer and shorter uteri had lower success rates [11]. We confirm that uterine length is an anatomical factor with a significant positive effect on IVF/ICSI success. Besides age and uterine length, catheter choice was independently associated with OPR and LBR with the soft Wallace catheter yielding a higher success rate than the rigid Labotect catheter. This is in accordance with data in the literature. For example, in a systematic review and meta-analysis of ten randomized trials, soft catheters were associated with a significantly higher pregnancy rate compared to firm catheters [2]. In our study, catheter choice was the only modifiable factor of ELT/TIEL-ET influencing the success rate of IVF/ICSI in our prospective study. Age and uterine length are given, whereas catheter choice is at the discretion of the treating physician. Thus, efforts should be made to preferentially use soft transfer catheters for ET. Of note, both catheter types were equally successful in terms of transferring the embryo at first attempt. Therefore, the higher OPR and LBR after the use of a soft catheter do not come at the price of a higher primary transfer failure rate as suggested previously [12].

Our study has strengths and weaknesses. First, this was a prospective cohort study and the times of ELT and TIEL-ET were exactly measured in a pre-defined way. This study design ensures a high data quality for ELT and TIEL-ET. Also, the study population is large and allows for a meaningful multivariate analysis. As compared to age of the patient or uterine length, ELT and TIEL-ET are controllable and may be used to increase the success rate of IVF/ICSI. Thus, the question of a possible prognostic value of the procedural speed of ELT and TIEL-ET is clinically relevant although it has received little attention in the literature. We did not randomize our patient population in slow vs fast ELT/TIEL-ET, because the time of ELT and TIEL-ET is a continuous variable without a biologically reasonable cut-off. Therefore, we have chosen a prospective observational study design. We think that this is the most appropriate study design to analyze the prognostic value of ELT and TIEL-ET.

It is a limitation of our study that we did not control for catheter choice and the individual person performing ELT and TIEL-ET, i.e., the technician and the physician. All study participants (technicians and physicians) were at an expert level, meaning that they all had a personal history of >100 ETs. Therefore, the external validity of our study only applies to expert performers. We cannot rule out that ELT and TIEL-ET might affect IVF/ICSI success among technicians and physicians with other levels of expertise. This has to be acknowledged when interpreting the results of our study. In addition, very long transfer times such as those described by Matorras et al. may compromise pregnancy rates [9]. Since almost all of the transfers described in our study were within the limit of 120 s described by Matorras, data of our study cannot be extrapolated to very long transfer times.

## Conclusion

In summary, we performed a prospective cohort study to assess the influence of ELT and TIEL-ET on the success of IVF/ICSI, defined as OPR and LBR. We found that both parameters were not associated with OPR and LBR in a univariate and multivariate analysis. These data demonstrate that speed of embryo transfer is not critical for the success of IVF/ICSI.
